# March of the Eosinophils: A Case of Eosinophilic Gastroenteritis, Immune Thrombocytopenia, and Iron Deficiency Anemia

**DOI:** 10.7759/cureus.28814

**Published:** 2022-09-05

**Authors:** Gurdeep Singh, Aimen Farooq, Arooj Mian, Baha Aldeen Bani Fawwaz, Peter Gerges, Abu H Khan

**Affiliations:** 1 Internal Medicine, AdventHealth Orlando, Orlando, USA; 2 Gastroenterology and Hepatology, AdventHealth Orlando, Orlando, USA

**Keywords:** atopy, iron deficiency anemia, immune thrombocytopenia, gastroenteritis, eosinophils

## Abstract

Eosinophilic gastrointestinal disorders (EGIDs) refer to eosinophilic infiltration of various sections of the gastrointestinal tract in the absence of secondary causes. Diagnosis of EGID requires histological evidence of eosinophilic infiltration of the GI tract. Here, we present a case of a young male with biopsy-proven eosinophilic gastroenteritis with a concomitant established diagnosis of immune thrombocytopenic purpura (ITP).

Presently, EGIDs remain an underexplored clinical entity. While its pathophysiology is not fully understood at this time, TH2 mediated activation of B-cells and subsequent stimulation of eosinophils locally appears to be at play. This is in contrast to the TH1 predominant cytokine profile underlying ITP, which this patient also has. Treatment typically involves dietary modifications and glucocorticoids.

## Introduction

Eosinophilic gastrointestinal disorders (EGIDs) refer to eosinophilic infiltration of various sections of the gastrointestinal tract in the absence of secondary causes [1]. Eosinophilic esophagitis has gained traction in recent years as a common cause of esophageal dysphagia in young adults, however eosinophilic gastritis (EG) and eosinophilic gastroenteritis (EGE) remain rarer clinical entities. The standardized prevalence of EG and EGE in the US was calculated to be around 6.3 per 100,000 and 8.4 per 100,000, respectively [2]. Clinical manifestations of EGIDs vary depending on the section of the GI tract involved, as well as the layer of tissue that has been infiltrated by eosinophils [3]. A diagnosis of EGE requires endoscopic evaluation and histopathological examination of gastric and intestinal tissue [3]. 

Here, we present a case of a young male with biopsy-proven eosinophilic gastroenteritis with a concomitant established diagnosis of immune thrombocytopenic purpura (ITP). 

## Case presentation

A 16-year-old male with a past medical history significant for autism, chronic ITP diagnosed at age 11, melanoma, allergic rhinitis, and eosinophilic esophagitis presented to the hospital for evaluation of chronic abdominal pain. The patient reported having various GI symptoms since he was three years old. Beyond abdominal pain, he noted bowel movements alternating between diarrhea and constipation. He also struggled with symptoms of acid reflux for which he was taking a proton-pump inhibitor. The patient had an extensive family history of allergies and atopic disease, including allergic rhinitis, asthma, and atopic dermatitis. 

The patient had undergone an upper endoscopy with a pediatric gastroenterologist that revealed findings consistent with eosinophilic esophagitis. He was advised to abstain from cow's milk after the allergy testing returned positive for dairy. However, the patient’s complaint of abdominal pain continued to persist over the course of the year. 

The patient ultimately underwent a repeat esophagogastroduodenoscopy (EGD) with biopsy that revealed duodenal mucosa with focal mucosal eosinophilia, severe mucosal eosinophilia, and reactive changes in the gastric body, fundus, and antrum, and squamous esophageal mucosa with mild inactive esophagitis and low-grade mucosal eosinophilia (up to 15 per high power field). Immunohistochemical staining for Helicobacter pylori was negative. The absolute eosinophil count at the time was 2542/uL. The patient was advised to refrain from dairy, egg, seafood, peanuts, wheat, and soy.

**Figure 1:**
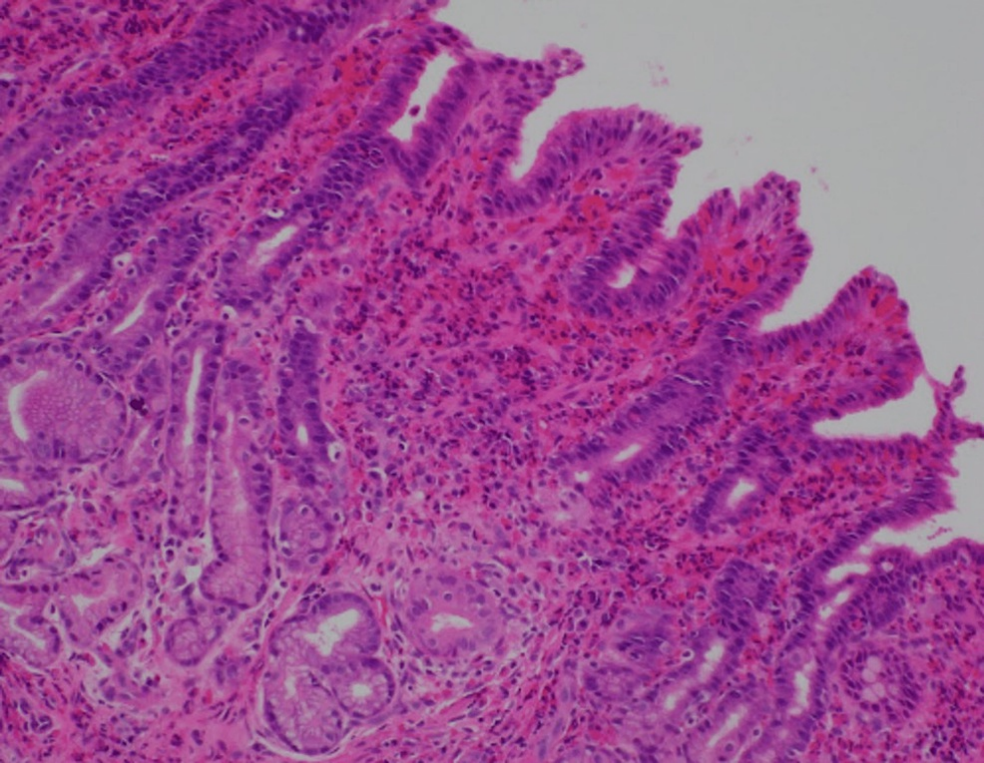
Cross-section of gastric tissue stained with hematoxylin and eosin demonstrating prominent eosinophils (greater than 100 per high-power field) within the lamina propria and gastric epithelium with associated epithelial injury.

The patient underwent repeat allergy testing twice that ultimately returned negative for food allergies but did reveal multiple environmental allergies. A repeat EGD performed one year later revealed white exudates at the level of the gastroesophageal junction, scattered erosions in the gastric body and fundus, as well as severe antral gastritis with multiple ulcers, erosions, and “pseudo-polypoid” lesions. Scattered erosions were seen throughout the duodenal bulb with no gross lesions identified in the distal duodenum. Biopsies were taken and revealed persistent distal eosinophilic esophagitis with greater than 50 eosinophils/HPF, prominent eosinophilic gastritis with greater than 100 eosinophils/HPF (Figure 1), chronic inflammation of the duodenal bulb with Brunner gland hyperplasia, focal foveolar cell metaplasia, and mild villous blunting. Absolute eosinophil counts consistently remained higher than 2,000/uL. Given findings of persistent inflammation despite a six-food elimination diet, the patient was started on oral budesonide along with a proton-pump inhibitor and H2 receptor blocker. 

Surveillance EGD performed six months later revealed resolution of esophagitis and duodenal inflammation. Previous findings of duodenal metaplasia and villous blunting had resolved, as did the polypoid lesions noted in the gastric mucosa. Roughly 28 eosinophils/HPF were present in the gastric cardia near the gastroesophageal junction (GEJ), and numerous eosinophils remained in the other gastric tissues sampled. The patient’s symptoms gradually improved, and oral budesonide was ultimately tapered and discontinued. 

On a follow-up visit three years later for surveillance EGD, the patient was found to have microcytic anemia, low folate, and iron levels, believed to be secondary to malabsorption caused by EGE. A diagnostic colonoscopy revealed no acute pathology in the colon or terminal ileum. EGD demonstrated persistent, diffuse eosinophilic gastritis involving the cardia, antrum, fundus, and body with minimal involvement of the distal esophagus (3 eosinophils/HPF) and no duodenal involvement. He was started on supplemental iron and folate, leading to resolution of the anemia; the patient currently remains on famotidine and pantoprazole with a plan for surveillance EGD every two to three years. 

## Discussion

Eosinophilic gastroenteritis is a rare clinical entity characterized by eosinophilic infiltration of the stomach and small intestine. The exact mechanism of EGE remains poorly understood. Jaffe et al. performed flow cytometry on three individuals with eosinophilic gastroenteritis and found normal levels and activity of CD4 and CD8 T-cells [4]. They noted an enhanced production of IL-4 and IL-5, two cytokines intimately involved in stimulating IgE synthesis and eosinophil activation, respectively [4]. These findings were echoed by Caldwell et al., who discovered increased expression of IL-4, IL-5, and IL-13 [5]. Local eotaxin-2 (CCL26) expression was also significantly increased in tissue from patients with EG compared to control tissue samples and was correlated with gastric tissue eosinophil counts [5]. Their findings in totality suggest that IL-13-mediated TH2 cell activation plays a role in EG/EGE [4,5]. 

Food allergy has also been suspected to play a role, as food allergy free diets have demonstrated an improvement in disease activity [6]. It has been reported that up to 50% of individuals with EGID have a concomitant food allergy or history of atopy [3,6]. Other findings that suggest a diagnosis of EGID include peripheral eosinophilia, elevated serum IgE levels, and findings compatible with malabsorption such as iron deficiency anemia, hypoalbuminemia, increased fecal fat elastase, abnormal liver function tests, prolonged prothrombin time (PT)/international normalized ratio (INR), and an abnormal D-xylose test [7]. 

Clinical manifestations of EGIDs vary depending on the section of the GI tract involved, as well as the layer of tissue that has been infiltrated by eosinophils [3]. EGE traditionally presents with nonspecific symptoms such as nausea, vomiting, abdominal pain, diarrhea, and weight loss [7]. Malabsorption can also be seen if there is involvement of the small intestine and can lead to various nutritional deficiencies [3,8]. Involvement of the muscular layer has led to cases of impaired gut motility and obstructive symptoms. Abdominal ascites has been reported in patients with EGID in the setting of subserosal involvement [3,7,8].

The diagnosis of EGE hinges on establishing eosinophilic inflammation of the stomach and small intestine, the absence of secondary causes of eosinophilia, and the lack of another organ involvement [1,6]. The presence of peripheral eosinophilia may suggest a diagnosis of EGE but is not required as up to 20% of patients with EGE have normal eosinophil levels in the blood [8,9]. Endoscopy and histologic examination of tissue samples obtained from a biopsy of the suspected sites remain the most reliable means of diagnosis [3,8]. A diagnosis of EGE is made when abnormally high numbers of eosinophils are found on microscopic examination of the stomach and duodenum [3,6,8]. The criteria for normal eosinophil count per HPF vary depending on the section of the GI tract being evaluated [10]. While there is no consensus value for how many eosinophils per HPF are required to diagnose EGE, pathologists such as Dr. Collins at Cincinnati Children’s Hospital recommend using 30/HPF when evaluating the duodenum and 30/HPF in five samples when evaluating the stomach [10]. Others, such as Gaballa et al., use a cutoff of 20 eosinophils/HPF to diagnose EGE [1,6,8,11,12]. 

Dietary modification as a means of treatment for EGE has been explored. Lucendo et al. performed a systematic literature review and found over 75% of children with EGE demonstrated clinical improvement with adherence to an elemental diet [13]. However, they note that evaluation was limited as histologic improvement was not assessed [13]. Furthermore, there was marked heterogeneity in the methodology of the studies they analyzed [13]. As such, they could not recommend the adoption of an elemental or empiric elimination diet as a viable means of treating EGE due to the lack of applicable data. Rached and Hajj endorse the use of a targeted elimination diet, where only food allergens detected on allergy testing are eliminated from the patient’s diet [1]. 

The mainstay pharmacotherapy for EGE presently is glucocorticoids [1,7,11]. Rached and Hajj recommend initiation of prednisone as the first-line choice for EGE as their literature review has revealed remission rates ranging anywhere between 50-90% [1]. If prednisone cannot be tolerated due to side effects, budesonide remains a viable alternative as it has a lower systemic side effect profile [1]. Other agents like azathioprine, cromolyn sodium, ketotifen, montelukast, and biologics like mepolizumab have been explored as adjuncts for the refractory disease but cannot be firmly recommended as first-line therapies due to conflicting data and lack of large-scale studies [1,14]. 

Immune thrombocytopenic purpura is an autoimmune condition in which autoantibodies that target platelets are formed, leading to their destruction. A relationship between EGID and immune thrombocytopenic purpura has yet to be established [8]. A literature review has revealed only one other case of EG and ITP, a 70-year-old woman from Europe. In their case report, Pan et al. recognized aberrant T-cell activation leading to B-cell stimulation and antibody production was the etiology underlying ITP [8]. However, Ogawara et al. and Noriyuki et al. were able to identify that ITP is associated with a high TH1/TH2 ratio [15,16]. Given that much of the available literature surrounding EGID suggests a TH2-mediated response, it presently remains unclear if there a causal association exists between the two. Further studies are needed into the presence of EGID and the development of concomitant autoimmune diseases, or vice versa. 

## Conclusions

Eosinophilic gastrointestinal diseases remain a rare and underexplored clinical entity. While its pathophysiology is not fully understood at this time, TH2-mediated activation of B-cells and subsequent stimulation of eosinophils locally appears to be at play. Diagnosis of EGID necessitates histological evidence of eosinophilic infiltration of the GI tract in the absence of secondary causes of infiltration elsewhere. The role of dietary modification in the treatment of EGID requires further studies but is a reasonable initial treatment if known food allergens are identified, glucocorticoids remain the mainstay of pharmacotherapy at this time.
